# Off-pump minimally invasive coronary artery bypass grafting in patients with left ventricular dysfunction: the lampang experience

**DOI:** 10.3389/fsurg.2024.1324343

**Published:** 2024-01-19

**Authors:** Ryohei Ushioda, Aina Hirofuji, Dit Yoongtong, Boonsap Sakboon, Jaroen Cheewinmethasiri, Hiroyuki Kamiya, Nuttapon Arayawudhikul

**Affiliations:** ^1^ Cardiovascular and Thoracic Surgery Unit, Department of Surgery, Lampang Hospital, Lampang, Thailand; ^2^ Department of Cardiac Surgery, Asahikawa Medical University, Asahikawa, Japan

**Keywords:** mini-thoracotomy, off-pump coronary artery bypass (OPCAB), minimally invasive coronary artery bypass grafting (CABG), left ventricular dysfunction, coronary artery revascularization

## Abstract

**Introduction:**

The minimally invasive cardiac surgery off-pump coronary artery bypass (MICSOPCAB) is technically difficult; therefore, previous studies have indicated that MICSOPCAB should be contraindicated in patients with impaired left ventricular (LV) function. In this study, we investigated the feasibility of MICSOPCAB in patients with impaired LV function.

**Methods:**

The 226 patients underwent MICSOPCAB between August 2017 and September 2022. Our study defined impaired LV function as ejection fraction (EF) in echocardiography 40% or less. The patients were divided into Low EF group (*n* = 39) and Normal EF group (*n* = 187).

**Results:**

The Low EF group was in a more critical preoperative condition than Normal EF group (41.0% in the Low EF group vs. 14.4% in the Normal EF group; *p* < 0.001). For preoperative transthoracic echocardiography, LV end-diastolic diameter (5.5 ± 0.9 cm in the Low EF group vs. 5.0 ± 0.8 cm in the Normal EF group; *p* < 0.001) and LV end-systolic diameter (4.4 ± 1.0 cm in the Low EF group vs. 3.4 ± 1.0 cm in the Normal EF group; *p *< 0.001) were significantly larger in the Low EF group. No differences were found in the operative time (180 [160–240] min in the Low EF group vs. 205 [165–253] min in the Normal EF group; *p* = 0.231) and the median number of distal anastomoses (2 [1–2] in the Low EF group vs. 2 [1–3] in the Normal EF group; *p* = 0.073). Intensive care unit stay was longer in the Low EF group than in the Normal EF group (2 [1–2] in the Low EF group vs. 1 [1–2] in the Normal EF group; *p* = 0.010). Perioperative transfusion was more common in the Low EF group than in the Normal EF group (69.7% vs. 49.2%; *p* = 0.023). There were no differences in major complications, hospital stay, and 30-day mortality. The Kaplan–Meier curve showed no significant difference in postoperative major adverse cardiac or cerebrovascular events rates between the two groups (*p* = 0.185)

**Conclusion:**

In this study, MICSOPCAB can be performed in patients with low EF having short- and mid-term outcomes similar to patients with normal EF. Therefore, low EF should not be contraindicated in MICSOPCAB.

## 1 Introduction

Recently, minimally invasive cardiac surgery (MICS) via small thoracotomy has been developed mainly in valvular surgery, and this trend is also seen in coronary artery bypass grafting [MICS off-pump coronary artery bypass (MICSOPCAB)]. MICSOPCAB has advantages over conventional off-pump coronary artery bypass (OPCAB) via median sternotomy, e.g., better cosmetics, less bleeding, and rapid recovery (1, 2). However, MICSOPCAB is technically difficult; therefore, previous studies have indicated that MICSOPCAB should be contraindicated in patients with impaired left ventricular (LV) function (3–5). Our institute has performed MICSOPCAB as a standard procedure in selected patients with favorable coronary anatomy regardless of LV function. In this study, we investigated the feasibility of MICSOPCAB in patients with impaired LV function.

## 2 Patients and methods

From August 2017 to September 2022, in a single institution, 226 patients underwent OPCAB via the left anterior mini-thoracotomy (8–10 cm) approach. Our MICSOPCAB project started with a single bypass of the left internal thoracic artery (LITA) to the left anterior descending artery (LAD) and has developed into a step-by-step multivessel bypass. In this study, impaired LV function was defined as LV ejection fraction (LVEF) in echocardiography of ≤40%, and MICSOPCAB included a single bypass. The patients were divided into Low EF group (*n* = 39) and Normal EF (*n* = 187).

### 2.1 Inclusion and exclusion criteria

All patients with favorable coronary targets presented from coronary angiogram deemed grafted upon a left anterior thoracotomy and normal or mild cardiomegaly from chest X-p from the heart team conference were considered for MICSOPCAB and included in the study. In a few patients, where complete revascularization of the right coronary system was not technically possible via a MICS approach, our heart team proposed hybrid coronary revascularization (HCR).

The principal exclusion criteria for MICSOPCAB consisted of associated mitral valvular dysfunction (more than moderate regurgitation), severe distal runoff of the coronary target, congenital heart disorders, severe chest deformities such as pectus excavatum and scoliosis, a recent history of stroke (4 weeks before surgery), chronic obstructive pulmonary disease, and combined aortic aneurysm (diameter >4 cm). Other contraindications included the history of tuberculosis or interstitial lung disease, active smoking, morbid obesity, and history of cardiac surgery. Our center has performed OPCAB surgery for >2,000 cases as a default operation with a conversion rate of <1% in the last 5 years; thus, acute myocardial infarction (MI) within the last 7 days, LVEF <20%, and cardiomegaly [LV end-diastolic diameter (LVDd) >6.0 cm] are the main precautions.

Before discharge, coronary computed tomography angiography (CTA) was performed in all consented patients with creatinine levels <1.5 mg/dl a week after the surgery.

### 2.2 Surgical technique

**Figure 1:**
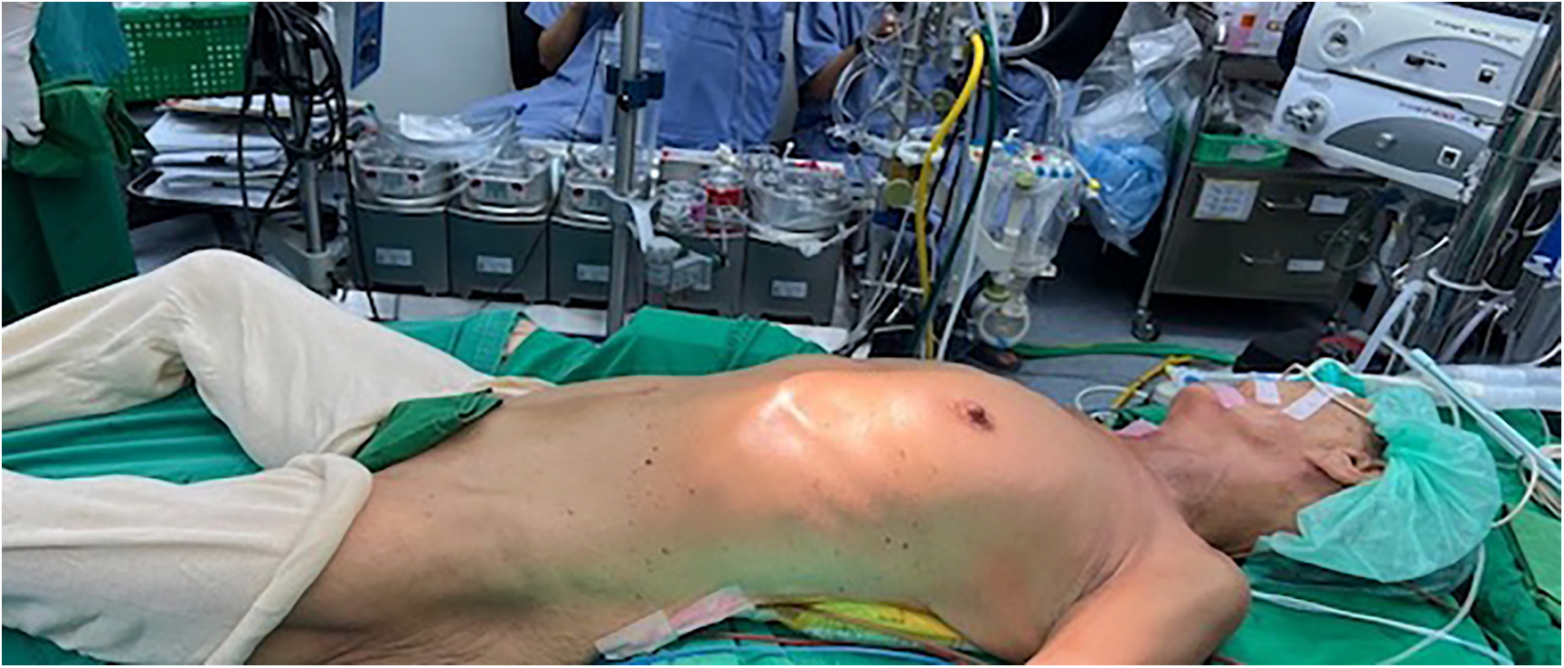
The patient was rotated 20–30 degrees to the left lateral position and intubated with a double-lumen endotracheal tube.

**Figure 2:**
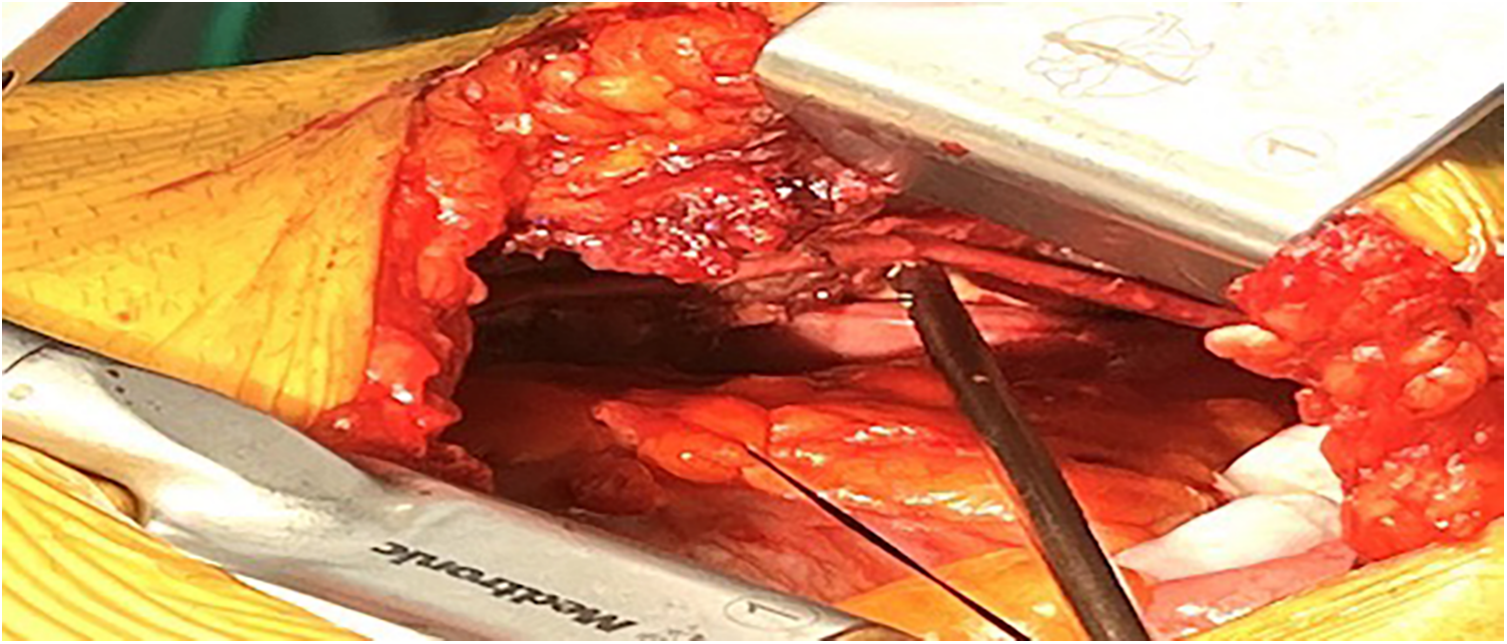
The internal thoracic artery skeletonized fashion under 8–10 cm left mini-thoracotomy in the fourth or fifth intercostal space.

The anesthesiologist and rehabilitation team assessed the patient's suitability for one-lung ventilation 1 or 2 days before the surgery. The patient was positioned by elevating the left chest 30°–40° with an inflatable bag behind to facilitate a widened intercostal space (ICS). The patients were intubated with a double-lumen endotracheal tube (Figure 1). The procedure was performed via an 8–10-cm left mini-thoracotomy (1/3 of the incision was medial to the mid-clavicular line) in the fourth or fifth ICS to access both internal thoracic arteries. A change in ICS depended on the degree of accessibility within the chest. For the best access to the left chest and optimally visualize the internal mammary arteries, the ThoraTrak® MICS Retractor System (Medtronic Inc., MN, USA) and the Skyhook Retractor System or Fehling MICS instruments (Fehling Instruments GmbH & Co., Germany) were used alternately (Figure 2). Nuvo Octopus R (TSMICS1, Medtronic Inc.), conventional Octopus R Evolution AS (TS2500, Medtronic Inc.), and deep pericardial stay sutures were used as cardiac stabilizers. The stabilizer or positioner type depended on the target exposure and hemodynamic stability; however, conventional Octopus and deep pericardial sutures were sufficient. Nevertheless, Nuvo Octopus was needed for the right internal thoracic artery (RITA) harvest or main pulmonary artery compression for proximal anastomosis on the ascending aorta. The entire LITA and RITA were skeletonized under direct vision. Saphenous vein grafts were mainly skeletonized with skip incisions. To maintain the activated clotting time >280 s, heparin (1 mg/kg) was administered after the internal thoracic artery (ITA) harvest. The standard sequence of anastomosis was the LAD, obtuse marginal (OM) branch, and last posterior descending artery (PDA), or posterolateral artery (PLA). To expose the lateral and inferior walls of the heart well, two deep pericardial stitches were routinely performed with the least hemodynamic compromise. Intracoronary shunt and CO_2_ Mister Blower (Medtronic Inc.) helped reduce the blood field during limited access, as with MICSOPCAB. Optimizing the heart's preload and maintaining a core temperature >34°C prevents decompensation and malignant arrhythmias. The on-pump conversion was considered if the patient's hemodynamics were unstable, or multiple ventricular arrhythmias were detected; however, there was 0% conversion in our series. Transit-time flow measurement (TTFM, MedistimR) was routinely used as a quality control for our procedures, and if there were any flaws, they were addressed and corrected before closing the chest. After protamine administration, chest drain number 28 was placed via the seventh ICS at the axillary line inside the left chest. Rib approximation was done by periosteal number 1 vicryl interrupted stitches, chest wall muscle with number 2 vicryl continuous stitches, and skin closure with 4/0 vicryl. Moreover, 20 ml of 0.5% Marcain was routinely infiltrated subcutaneously to control postoperative pain.

### 2.3 Postoperative management

After extubation, each patient received dual antiplatelets (one tablet of aspirin 80 mg and clopidogrel 75 mg) and 2–3 mg intravenous morphine for pain management. Subsequently, they received paracetamol (500 mg) four times daily and Tylenol with codeine (15 mg) three times daily. Postoperative pain was assessed using the Wong–Baker Faces pain-rating scale. If the pain score was >8, additional optimal doses of morphine or fentanyl were given intravenously (using a rate of about 30%). The intercostal drainage was removed if the total drain was <100 ml over 24 h.

Dual antiplatelet therapy, an H_2_ blocker, and statin were prescribed as discharge medications. Ticagrelor (180 mg) was also used alternately with clopidogrel in previously implanted drug-eluting stents or the HCR group. When hemodynamics became stable, lifelong amlodipine 5 mg/day was prescribed for those who underwent radial artery grafting.

### 2.4 Follow-up

Follow-up information of all patients was actively collected through the planned outpatient clinic. The mean follow-up duration was 677.0 ± 517.3 days, with a follow-up rate of 100%.

### 2.5 Statistical analysis

The t-test was used for continuous variables with normal distribution, whereas the Mann–Whitney *U*-test was used for those with non-normal distribution. Fisher's test was utilized to analyze categorical variables. Statistical significance was set at *p* < 0.05. The Kaplan–Meier method demonstrated an absence of major adverse cardiac or cerebrovascular events (MACCE). A multivariable Cox regression analysis was used to identify independent factors of MACCE, which was presented as the hazard ratio (HR) with 95% CI. The STATA Software/MP, Version 17.0 (Stata Corporation, College Station, Texas, USA) was used for the statistical analyses.

## 3 Results

The demographic characteristics of both groups are summarized in Table 1. The average age of the patients was 64.5 ± 10.0 years in the Low EF group and 64.6 ± 8.1 years in the Normal EF group (*p *= 0.961). The male ratio was higher in the Normal EF group (48.7% in the Low EF group vs. 66.1% in the Normal EF group; *p* = 0.040). Low EF group was in a more critical preoperative condition than Normal EF group (41.0% in the Low EF group vs. 14.4% in the Normal EF group; *p *< 0.001). The preoperative patient condition was worse in the Low EF group: New York Heart Association (NYHA) (48.7% in the Low EF group vs. 12.3% in the Normal EF group; *p* < 0.001), Euro SCORE II (2.90 [1.62–6.15] in the Low EF group vs. 1.3 [0.87–2.10] in the Normal EF group; *p* < 0.001), and preoperative intra-aortic balloon pumping (IABP) (10.3% in the Low EF group vs. 2.7% in the Normal EF group; *p *= 0.050), reflecting impaired LV function. For preoperative transthoracic echocardiography, LVDd (5.5 ± 0.9 cm in the Low EF group vs. 5.0 ± 0.8 cm in the Normal EF group; *p* < 0.001) and LV end-systolic diameter (LVDs; 4.4 ± 1.0 cm in the Low EF group vs. 3.4 ± 1.0 cm in the Normal EF group; *p* < 0.001) were significantly larger in the Low EF group. This study did not include severe Low EF cases under 20% (31.9 ± 6.5% in the Low EF vs. 59.1 ± 11.1% in the Normal EF group; *p* < 0.001). In the Normal EF group, HCR was performed in six patients with preoperative percutaneous coronary intervention in the right coronary artery, whereas no patient in the Low EF group underwent HCR (*p* = 0.593).

**Table 1:** Patients’ characteristics and preoperative data.

Variables	Over all (*n* = 226)	Low EF group (*n* = 39)	Normal EF group (*n* = 187)	*p*-value
Age, mean ± SD years	64.7 ± 8.5	64.5 ± 10.0	64.6 ± 8.1	0.961
Male gender, *n* (%)	142 (62.8)	19 (48.7)	123 (66.1)	0.040
Weight, mean ± SD kg	58.4 ± 11.8	56.5 ± 11.5	58.8 ± 11.8	0.286
Height, mean ± SD cm	159.5 ± 7.7	157.2 ± 6.2	159.9 ± 7.9	0.046
NYHA class (≧Ⅲ), *n* (%)	42 (18.6)	19 (48.7)	23 (12.3)	<0.001
Euro SCORE, median [IQR]	1.41 [0.90–2.59]	2.90 [1.62–6.15]	1.3 [0.87–2.10]	<0.001
Comorbidity, *n* (%)				
Hyperlipidemia	215 (95.1)	39 (100)	176 (94.1)	0.219
Hypertension	218 (96.5)	37 (94.9)	181 (96.8)	0.629
Diabetes mellitus	101 (44.7)	22 (56.4)	79 (42.2)	0.106
Chronic renal disease (Cr≧1.5)	30 (13.3)	6 (15.4)	24 (12.8)	0.669
COPD	16 (7.0)	3 (7.7)	13 (7.0)	0.743
Smoker	62 (27.4)	11 (28.2)	51 (27.4)	0.920
Cerebral vascular accident	14 (6.2)	3 (7.7)	11 (5.9)	0.714
PAD	22 (9.7)	6 (15.4)	16 (8.6)	0.231
STEMI	42 (18.6)	11 (28.2)	31 (16.6)	0.089
Recent MI	122 (54.0)	26 (66.7)	96 (51.3)	0.081
SVD	42 (18.6)	12 (30.8)	30 (16.0)	0.032
DVD	98 (43.4)	17 (43.3)	81 (43.3)	0.975
TVD	86 (38.1)	10 (25.6)	76 (40.1)	0.079
Preoperative PCI	26 (11.5)	4 (10.3)	22 (11.8)	1.000
HCR	6 (2.7)	0 (0)	6 (3.2)	0.593
Preoperative IABP	9 (4.0)	4 (10.3)	5 (2.7)	0.050
Echocardiography				
LVDd, mean ± SD mm	5.1 ± 0.9	5.5 ± 0.9	5.0 ± 0.8	<0.001
LVDs, mean ± SD mm	3.6 ± 1.1	4.4 ± 1.0	3.4 ± 1.0	<0.001
LVEF, mean ± SD %	54.5 ± 14.7	31.9 ± 6.5	59.1 ± 11.1	<0.001
Urgency, *n* (%)				
Elective	181 (80.1)	22 (56.4)	159 (85.0)	<0.001
Urgent	41 (18.1)	16 (41.0)	25 (13.4)	<0.001
Emergent	1 (0.4)	0 (0)	1 (0.5)	1.000
Salvage	3 (1.3)	1 (2.6)	2 (1.1)	0.435

NYHA, New York Heart Association; COPD, chronic obstructive pulmonary disease; PAD, peripheral arterial disease; STEMI, ST-elevation myocardial infarction; SVD, single vessel disease; DVD, double vessel disease; TVD, triple vessel disease; PCI, percutaneous coronary intervention; HCR, hybrid coronary revascularization; IABP, intra-aortic balloon pumping; LVDd, left ventricular end-diastolic diameter; DVDs, left ventricular end-systolic diameter; LVEF, left ventricular ejection fraction.

**Figure 3:**
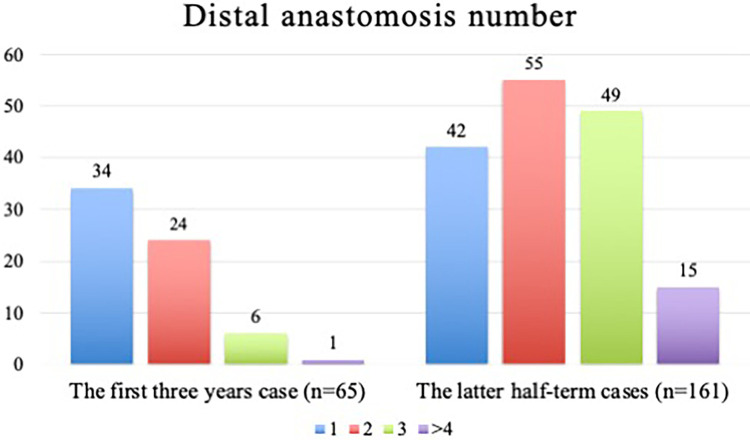
The comparison of an average number of distal anastomoses between the first (*n* = 65) and later three years (*n* = 161).

Operative data are shown in Table 2. No differences were found in the operative time (180 [160–240] min in the Low EF group vs. 205 [165–253] min in the Normal EF group; *p* = 0.231) and the median number of distal anastomoses (2 [1–2] in the Low EF group vs. 2 [1–3] in the Normal EF group; *p* = 0.073). The comparison of an average number of distal anastomoses between the first (*n* = 65) and later 3 years (*n* = 161) is shown in Figure 3. The number of grafts was significantly higher in Normal EF group (1 [1–2] in the Low EF group vs. 2 [1–3] in the Normal EF group; *p* = 0.008). The total and multiple arterial graft proportions showed no significant differences between groups (15.4% and 23.0% in the Low EF group vs. 23.0% and 25.7% in the Normal EF group; *p* = 0.294 and 0.171, respectively). Intraoperative conversion to median sternotomy occurred in two patients (2.6% in the Low EF group vs. 0.5% in the Normal EF group, *p* = 0.218) because both patients had ITA injuries. No patients required intraoperative IABP and on-pump conversion.

**Table 2:** Operative data.

Variables	Over all (*n* = 226)	Low EF group (*n* = 39)	Normal EF group (*n* = 187)	*p*-value
Operating time, median [IQR] min	200 [165–249]	180 [160–240]	205 [165–253]	0.231
Total grafts numbers, median [IQR]	2 [1–2]	1 [1–2]	2 [1–3]	0.008
The number of distal anastomoses median [IQR]	2 [1–3]	2 [1–2]	2 [1–3]	0.073
Sequential technique, *n* (%)	53 (23.4)	7 (18.0)	46 (24.6)	0.373
Y-composite graft, *n* (%)	75 (33.2)	8 (20.5)	67 (35.8)	0.065
Complete revascularization, *n* (%)	145 (64.2)	26 (66.7)	119 (63.6)	0.720
Conversion to sternotomy, *n* (%)	2 (0.9)	1 (2.6)	1 (0.5)	0.316
Graft, *n* (%)				
LITA	221 (97.8)	38 (97.4)	183 (97.9)	0.870
RITA	18 (8.0)	3 (7.7)	15 (8.0)	0.945
Bilateral ITA	15 (6.6)	2 (5.2)	13 (7.0)	0.677
Radial artery	43 (19.0)	5 (12.8)	38 (20.3)	0.278
Gastroepiploic artery	9 (4.0)	1 (2.6)	8 (4.3)	0.619
Total arterial graft	49 (21.7)	6 (15.4)	43 (23.0)	0.294
Multiple arterial grafts	54 (23.9)	6 (15.4)	48 (25.7)	0.171
Saphenous vein	106 (46.9)	13 (33.3)	93 (49.7)	0.062
Distal anastomoses, *n* (%)				
Left anterior descending artery	224 (99.1)	39 (100)	185 (98.9)	0.517
Diagonal branch	19 (8.4)	2 (5.19)	17 (9.1)	0.417
Ramus intermedius	12 (5.3)	2 (5.19)	10 (5.3)	0.956
Obtuse marginal branch	129 (57.1)	18 (46.2)	111 (59.4)	0.130
Posterior descending artery	58 (25.7)	5 (12.8)	53 (28.3)	0.044
Posterior branch of the left ventricle	9 (4.0)	0 (0)	9 (4.8)	0.162

LITA, left internal thoracic artery; RITA, right internal thoracic artery; BITA, bilateral internal thoracic artery.

Short-term outcomes are shown in Table 3. Intensive care unit (ICU) stay was longer in the Low EF group than in the Normal EF group (2 [1–2] in the Low EF group vs. 1 [1–2] in the Normal EF group; *p* = 0.010). Perioperative transfusion was more common in the Low EF group than in the Normal EF group (69.7% vs. 49.2%; *p* = 0.023). On the contrary, except for new-onset atrial fibrillation, no differences were found in postoperative complications, hospital stay, 30-day mortality, and patency rates of all grafts of the CTA (90.0% in the Low EF group vs. 90.3% in the Normal EF group; *p* = 0.175). The patency rates for each coronary artery target were as follows: LAD, 96.9% (96.6% in the Low EF group vs. 97.5% in the Normal EF group; *p* = 0.052); diagonal branch, 100%; ramus intermedius, 100%; OM, 94.1% (93.7% in the Low EF group vs. 95.0% in the Normal EF group; *p* = 0.594); PDA, 87.0% (75.0% in the Low EF group vs. 88.0% in the Normal EF group; *p* = 0.436); and PLA, 90.0% (0% in the Low EF group vs. 90.0% in the Normal EF group; *p* = 1.000).

**Table 3:** Postoperative short-outcomes.

Variables	Over all (*n* = 226)	Low EF group (*n* = 39)	Normal EF group (*n* = 187)	*p*-value
ICU stay, median [IQR]days	1 [1–2]	2 [1–2]	1 [1–2]	0.010
Hospital stay, median [IQR]days	5 [5–6]	5 [5–6]	5 [5–6]	0.989
Early extubation (≦24 h), *n* (%)	206 (91.2)	34 (87.2)	172 (92.0)	0.353
Perioperative transfusion, *n* (%)	119 (52.7)	27 (69.2)	92 (49.2)	0.023
Drain contents, Median [IQR] (ml)	320 [250–500]	400 [250–500]	300 [250–460]	0.370
Postoperative peak troponin I, Median [IQR] (ng/L)	1,119 [428–3,037]	2,012 [751–5,816]	1,148 [412–2,728]	0.074
30-day mortality, *n* (%)	3 (1.3)	1 (2.6)	2 (1.1)	0.435
Postoperative complications, *n* (%)				
New stroke	0 (0)	0 (0)	0 (0)	
New Dialysis	0 (0)	0 (0)	0 (0)	
New on-set atrial fibrillation	51 (22.6)	16 (41.0)	35 (18.7)	0.002
Reintubation	2 (0.9)	0 (0)	2 (1.1)	1.000
Infection	0 (0)	0 (0)	0 (0)	
Reoperation of bleeding	6 (2.7)	2 (5.1)	4 (2.1)	0.277
Postoperative CTA				
Patients with follow-up CTA, *n* (%)	195 (86.3)	30 (76.9)	165 (88.2)	0.074
Totalgraft patency, *n* (%)	176 (77.9)	27 (90.0)	149 (90.3)	0.175
LAD patency, *n* (%)		29 (96.6)	159 (97.5)	0.052
Diagonal branch patency, *n* (%)	16 (100)	1 (100)	15 (100)	
Ramus intermedius patency, *n* (%)	11 (100)	2 (100)	9 (100)	
OM branch patency, *n* (%)		15 (93.7)	96 (95.0)	0.594
PDA patency, *n* (%)		3 (75.0)	44 (88.0)	0.436
PL patency, *n* (%)		0 (0)	9 (90.0)	1.000

ICU, intensive care unit; CTA, computed tomography angiography; LAD, left anterior descending artery; OM, obtuse marginal; PDA, posterior descending artery; PL, postero-latateral artery.

**Figure 4:**
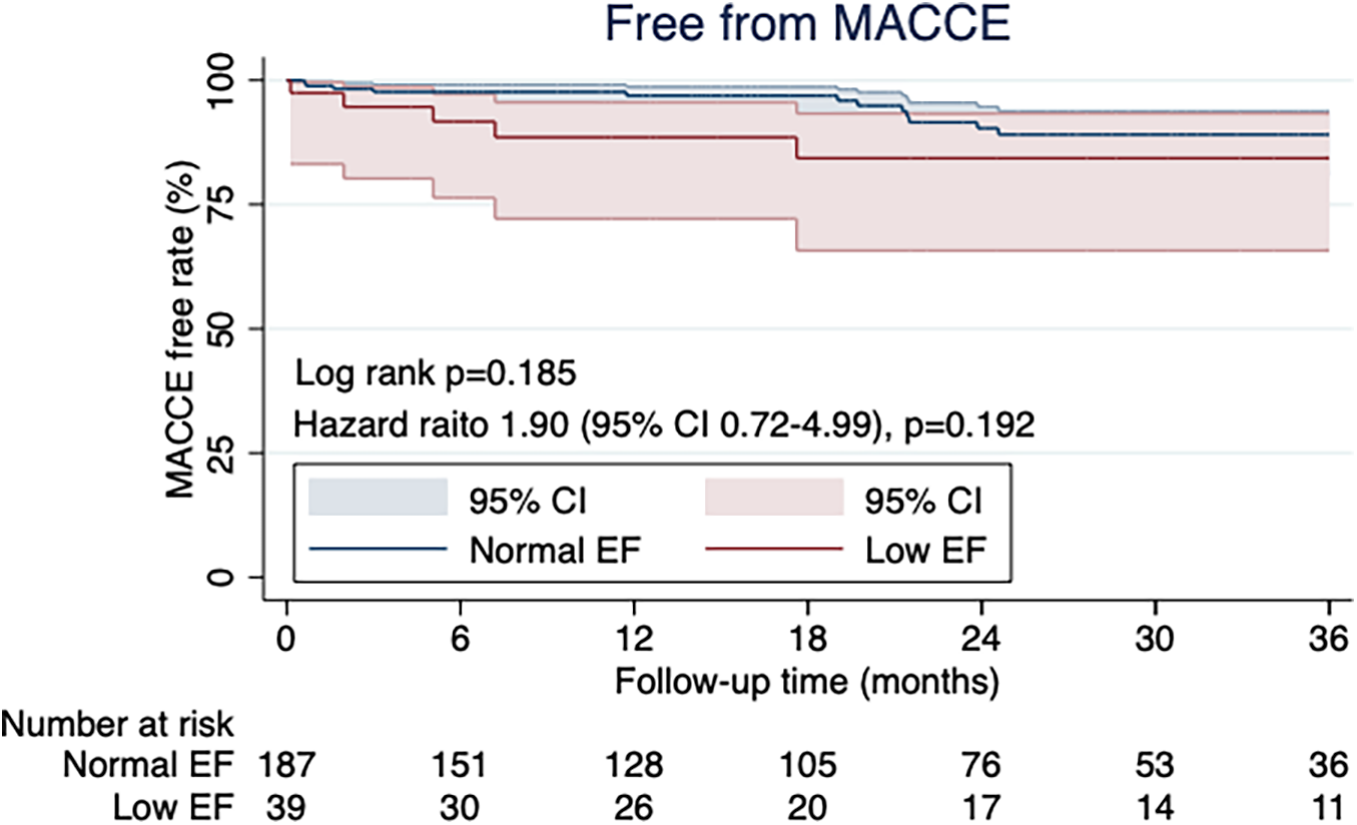
The kaplan-meier curve for free MACCE rate showed no significant differences between the two groups (*p* = 0.185).

The Kaplan–Meier curve showed no significant difference in postoperative MACCE rates between the two groups (*p* = 0.185; Figure 4). The types of MACCE, namely, cardiac death, repeat revascularization, heart failure requiring hospitalization, MI, and stroke in the follow-up periods, are shown in Table 4.

**Table 4:** Postoperative MACCE.

Variables	Over all (*n* = 226)	Low EF group (*n* = 39)	Normal EF group (*n* = 187)	*p*-value
Median follow-up days, [IQR]	596 [217–999]	653 [208–1,201]	592 [231–970]	0.782
MACCE, long-term, *n* (%)	20 (8.8)	6 (15.4)	14 (7.5)	0.125
Total cardiac death	8 (3.5)	2 (5.1)	6 (3.2)	0.629
Total mortality	13 (5.8)	5 (12.8)	8 (4.3)	0.053
Cerebral vascular accident	8 (3.5)	3 (7.7)	5 (2.7)	0.143
HF requiring hospitalization	2 (0.9)	0 (0)	2 (1.1)	1.000
Postoperative MI	5 (2.2)	0 (0)	5 (2.7)	0.591

MACCE, major adverse cardiac or cerebrovascular events; HF, heart failure; MI, myocardial infarction.

Multivariate logistic regression analyses were performed to test potential pre and intra-operative risk factors of MACCE (Table 5). The Hosmer–Lemeshow test indicates a good overall fit for the model employing Schoenfeld residuals (*p* = 0.0347). The Deviance test did not yield statistically significant results (*p* = 1.000). Established negative factors of MACCE (total arterial revascularization and complete revascularization) were not confirmed in our patient cohorts. Low EF patients were not risk factors specifically for MACCE (HR = 1.84, 95% CI: 0.70–4.85, *p* = 0.216).

**Table 5:** Multivariable analyses for factors associated with MACCE. Confirmation of well-described MACCE negative factors.

Variables	HR	95% CI	*p*-value
Age	1.03	0.98–1.09	0.782
Male gender	1.08	0.42–2.79	0.125
Low EF (under 40%)	1.84	0.70–4.85	0.629
Complete revascularization	1.22	0.38–3.87	0.053
Total arterial grafts revascularization	0.89	0.245–3.27	0.143

MACCE, major adverse cardiac or cerebrovascular events; EF, ejection fraction; EF, left ventricular ejection fraction.

## 4 Discussion

In this study, patients who underwent MICSOPCAB showed no significant difference in graft patency rate, 30-day mortality, and absence of MACCE in the mid-term follow-up between those with preserved and impaired LV function.

### 4.1 On-pump coronary artery bypass (ONCAB) vs. OPCAB in patients with impaired LV function

Coronary artery bypass grafting is effective and recommended by major guidelines for treating patients with low EF caused by ischemia (6–8). On the contrary, the choice of CABG modality, that is, ONCAB or OPCAB, in patients with low EF remains unclear; however, generally, ONCAB is preferred for LV dysfunction because of concerns about hemodynamic instability during heart positioning. By contrast, several studies have compared off-pump and on-pump CABG in patients with low EF, and the mid- and long-term outcomes were comparable for both procedures (5, 9–11). Attaran et al. reported the safety of OPCAB with an EF of <30% (5). In their study, 934 patients with low EF underwent isolated CABG (ONCAB group, *n* = 528; OPCAB group, *n* = 406). The length of ICU stay, hospital stay, and ventilation time were considerably shorter in the OPCAB group (*p* < 0.05), although mid-term (5 years) and long-term (10 years) survival rates were comparable with matched preoperative characteristics. The use of ONCAB vs. OPCAB in patients with impaired LV function is still controversial; however, our institution focuses on the benefits of OPCAB. These include lower incidence of postoperative stroke, perioperative transfusion, incomplete cardioplegic perfusion, and shorter hospital stays (5, 12, 13). Many patients in Thailand prefer shorter hospital stays because of their low socioeconomic status. Therefore, our first choice for patients with low EF is OPCAB.

However, previous studies in patients with low EF have reported that distal anastomoses were fewer in the OPCAB group than in the ONCAB group (13–15). Patients with low EF have cardiomegaly and lower tolerance against heart positioning; thus, OPCAB is sometimes difficult, particularly if the target vessels are located on the posterolateral wall. As previous studies have suggested, multivessel OPCAB in patients with low EF should be performed in high-volume centers with advanced experience and skilled surgeons.

### 4.2 Conventional OPCAB via median sternotomy vs. MICSOPCAB in patients with impaired LV function

Surgical revascularization through left thoracic minimal incision was popularized in the 1990s as minimally invasive direct coronary artery bypass grafting (MIDCAB) (16) and evolved to multivessel bypass. Currently, multivessel OPCAB via left small thoracotomy has been developed as MICSOPCAB and is gaining attention. However, whether MICSOPCAB can be safely performed in patients with impaired LV function compared with conventional OPCAB via median sternotomy remains unclear.

MICSOPCAB offers better cosmetics, less surgical trauma, and shorter hospitalization than OPCAB via median sternotomy; therefore, it may be attractive for patients if performed safely (1, 2). In addition to LV function, previous reports have demonstrated the noninferiority of MICAOPCAB to conventional OPCAB via median sternotomy (1, 2). Liang et al. demonstrated the safety of MICSOPCAB in their large-volume study with 582 patients (2). In their study, patients in the MICSOPCAB group were propensity score-matched with those in the OPCAB at a 1:1 ratio (MICSOPCAB = 172; OPCAB = 172), using epidemiological data, preoperative clinical characteristics, and SYNTAX score as covariates. MICSOPCAB was associated with a longer operative duration, higher postoperative hemoglobin levels and activities of daily living index values, and a shorter duration of postoperative hospitalization (*p* < 0.05). Moreover, no differences in 6-month graft patency were observed between the groups. Thus, MICSOPCAB may be performed safely, similar to OPCAB, via median sternotomy, albeit in limited high-volume centers. Regarding multivessel arterial grafting, MICSOPCAB is also evolving. Harvesting bilateral internal mammary arteries via left small thoracotomy has been challenging; however, their safety has improved in recent years (4, 17). This study used bilateral internal mammary arteries in 15 patients. With careful patient selection, evidence has indicated that MICSOPCAB can be performed safely without compromising the graft selection strategy.

However, as mentioned above, no study has examined the safety of MICSOPCAB in patients with impaired LV function; therefore, whether low EF should be included in patient selection is still unclear. In the present series, MICSOPCAB showed significant differences in the ICU stay (2 [1–2] in the Low EF group vs. 1 [1–2] in the Normal EF group; *p* = 0.010) and perioperative transfusions (69.7% in the Low EF group vs. 49.2% in the Normal EF group; *p* = 0.023) but did not compromise hospital stay or complication rates in patients with low EF, comparable to other recent studies (1, 2, 4, 17). Based on the results of the present study, we advocated that low EF should not be identified as an absolute contraindication for MICSOPCAB.

### 4.3 Technical tips and tricks for MICSOPCAB in patients with impaired LV function

MICSOPCAB requires special anesthesiologist and surgical attention because of the difficulty in maintaining hemodynamics during anastomosis (3). The surgeon typically begins with a single graft of the LIMA to the LAD in their MICSOPCAB path. In the present study, we also increased the distal anastomoses between the first (*n* = 65) and later 3 years (*n* = 161), as shown in Figure 3. In addition, these patients have limited working space because of cardiomegaly. In the present study, the heart was significantly enlarged in patients with low EF compared with other parameters (LVDd, 5.5 ± 0.9 cm in the Low EF group vs. 5.0 ± 0.8 cm in the Normal EF group, *p* < 0.001; LVDs, 4.4 ± 1.0 cm in the Low EF group vs. 3.4 ± 1.0 cm in the Normal EF group, *p* < 0.001). However, no intraoperative IABP addition or cardiopulmonary bypass was needed. Moreover, no significant difference in the median number of distal anastomoses (2 [1–2] in the Low EF groupvs. 2 [1–3] in the Normal EF group; *p* = 0.073) was found. We consider MICSOPCAB advantageous over conventional OPCAB because it does not require moving the heart much when making an anastomosis of the lateral and posterior walls due to a lateral chest wall incision. In our opinion, here are some tips and techniques for low EF cases: (1) avoid vigorous volume loading as in sternotomy, (2) make a spacious pericardial opening, (3) routinely apply intracoronary shunt, (4) start anastomosis from the LAD, (5) complete revascularization or hybrid revascularization, and (6) routinely use of TTFM.

### 4.4 Study limitations

This study has some limitations. First, this study is a retrospective, nonrandomized analysis from a single medical center. Second, all procedures were performed by one surgeon. Third, the median follow-up period was relatively short (653 days in the Low EF group vs. 597 days in the Normal EF group; *p* = 0.782). Third, after hospital discharge, follow-up coronary angiography was not generally performed because of the lack of insurance reimbursement for this procedure in Thailand. Still, it was considered for patients with clinical symptoms indicative of cardiac ischemia. Fourth, the two groups had different patient backgrounds, with more severe cases; for example, urgent cases (*p* < 0.001), high Euro SCORE Ⅱ (*p* < 0.001), NYHA ≧ Ⅲ (*p* < 0.001), and preoperative IABP (*p* = 0.050) in the Low EF group. Therefore, comparing the outcomes between the two groups may not be possible.

## 5 Conclusion

In this study, MICSOPCAB can be performed in patients with low EF having short- and mid-term outcomes similar to patients with normal EF. Therefore, low EF should not be contraindicated in MICSOPCAB.

## Data Availability

The datasets presented in this article are not readily available because the use of the dataset is primarily restricted to research purposes, and commercial use is not permitted. The dataset contains personal information, and handling it with consideration for privacy is required. Requests to access the datasets should be directed to RU, ryouheihei98@gmail.com.
